# Treatment of Diarrhɶa by Electricity

**Published:** 1893-08

**Authors:** 


TREATMENT OF DIARRHŒA BY ELECTRICITY. — Dr. Ervant Arslan, formerly assistant to Dr. Jules Simon at the Hospital des Enfants-Malades, of Paris, reports to the Societe de Biologie on the application of the faradic current directly to the abdominal wall in bowel disorders of children. In fifteen cases he had successfully resorted to this procedure with remarkable success in promptly controlling both diarrhœa and vomiting, no other remedies being given. In three cases of cholera in infants the electric current was used in this manner with complete success in each case. Two of them received at the same time other treatment, but the third, in whom the symptoms were the most grave, was subjected solely to electricity. The diarrhœa in all rapidly ceased, but the vomiting continued until the curreut was sent along the pneumogastric, by placing one electrode over the vasculo-nervous tract in the neck and the other upon the epigastrium for a minute or two at a time. The vomiting then ceased and did not recur. The general symptoms also began to ameliorate, and the patients were convalescent in three or four days.

The current from any induction apparatus will answer the purpose; it should be strong enough to produce contraction of the muscular fibres. The electrodes should be frequently moistened and applied in various locations over the abdominal surface. One application, early in the morning, each day, is usually sufficient in ordinary diarrhœa; in graver cases, and in cholera, the current may be applied twice in the twenty-four hours. In some cases a single application is sufficient to check the further progress of the malady; the diarrhœa may even be succeeded by constipation, as in one of the cases referred to.

In general, after three or four applications at most, the diarrhœa stops and other symptoms are mitigated.

Exceptions to this are found in diarrhœa due to dysentery and ulcerative entero-colitis. In other forms this method gives excellent results, even in the diarrhœa of phthisis, and this may be so held under control as to permit increase of nutrition and of the strength of the little sufferers. It is possible that this mode of treatment may also prove applicable to adults. It has, at least, the advantage of not interfering with other remedial or dietetic measures which may be deemed proper or necessary by the physician in charge of the case.

